# Cytotoxic and Antioxidant Potential of *Launaea mucronata* Forssk Essential Oil Growing in Northern Saudi Arabia

**DOI:** 10.3390/molecules28207025

**Published:** 2023-10-11

**Authors:** Ehab M. Mostafa, Ahmed H. El-Ghorab, Mohammed M. Ghoneim, Hasnaa Ali Ebrahim, Moaz Abulfaraj, Mohamed A. Abdelgawad, Amr Farouk, Arafa Musa

**Affiliations:** 1 Department of Pharmacognosy, College of Pharmacy, Jouf University, Sakaka 72341, Saudi Arabia; emmoustafa@ju.edu.sa; 2 Pharmacognosy and Medicinal Plants Department, Faculty of Pharmacy (Boys), Al-Azhar University, Cairo 11884, Egypt; 3 Chemistry Department, College of Science, Jouf University, Sakaka 72341, Saudi Arabia; 4 Flavour and Aroma Chemistry Department, National Research Centre, Dokki, Giza 12622, Egypt; af.mansour@nrc.sci.eg; 5 Department of Pharmacy, College of Pharmacy, AlMaarefa University, Riyadh 13713, Saudi Arabia; mghoneim@um.edu.sa; 6 Department of Basic Medical Sciences, College of Medicine, Princess Nourah bint Abdulrahman University, Riyadh 11671, Saudi Arabia; haebrahim@pnu.edu.sa; 7 Department of Surgery, Faculty of Medicine, King Abdulaziz University, Jeddah 21589, Saudi Arabia; mabolafaraj@kau.edu.sa; 8 Department of Pharmaceutical Chemistry, College of Pharmacy, Jouf University, Sakaka 72341, Saudi Arabia; mhmdgwd@ju.edu.sa

**Keywords:** *Launaea mucronata*, volatile oil, antioxidant, antiproliferative, docking, Asteraceae

## Abstract

Essential oils are naturally occurring multicomponent combinations of isoprenoids with distinctive odors that are produced by aromatic plants from mevalonic acid. They are extensively applied in aromatherapy for the treatment of various ailments. To investigate the potential therapeutic value of the ingredients in *Launaea mucronata* essential oil (EO), gas chromatography–mass spectrometry (GC-MS) analysis was used for essential oil characterization. Then, 2,2-diphenyl-1-picrylhydrazyl (DPPH), *β*-carotene/linoleic acid, and 2,2′-azino-bis(3-ethylbenzothiazoline-6-sulfonic acid) (ABTS) assays were used to evaluate the antioxidants. A 3-(4,5-dimethylthiazol-2-yl)-2,5-diphenyl-2H-tetrazolium bromide (MTT) assay was used to estimate the cytotoxicity. Following a thorough analysis of the GC-MS chromatogram, 87 components representing 97.98% of the entire EO mixture were identified. N-eicosane (10.92%), 2*E*,6Z-farnesol (10.74%), and 2*Z*,6*E*-farnesyl acetone (46.35%) were determined to be the major components of the oil. When the produced EO was evaluated for its antioxidant properties, it showed a strong inhibitory effect (%) of 65.34 at a concentration of 80 μg/mL. The results (g/mL) showed a positive response against the tested cell lines for HCT-116, MCF-7, and HepG2 (8.45, 10.24, and 6.78 g/mL, respectively). A high-concentration mixture of deadly components consisting of farnesol, bisabolol, eicosane, and farnesyl acetone may be responsible for this significant cytotoxic action, which was especially noticeable in the HepG2 cell line. Molecular docking occurred between farnesol and farnesyl acetone with the target residues of topoisomerases I and II, CDK4/cyclD1, and Aurora B kinases; these showed binding free energies ranging from −4.5 to −7.4 kcal/mol, thus demonstrating their antiproliferative action. In addition, farnesol and farnesyl acetone fulfilled most of the ADME and drug-likeness properties, indicating their activity.

## 1. Introduction

Aromatherapy is a well-known kind of complementary medicine that can be used through inhalation to treat a variety of conditions, including headache, nasal congestion, anxiety, and sleep disturbances [1,2,3]. To reduce tension, one can massage aromatherapeutic substances topically into the skin. Due to its total reliance on the use of essential oils (EOs), aromatherapy has been given the label “essential oil therapy”. Naturally occurring volatile distillates are dispersed throughout nature and are frequently responsible for the distinctive flavors and fragrances of various plants [2]. They are found in almost all plant organs, including fruits (citrus), flowers, leaves, roots, and stems [2]. 

Essential oils are applied for a variety of functions, such as attracting insects to help with flower pollination and seed dissemination or repelling unwelcome insects [4,5]. These substances are also significant parts of defense strategies due to their antibacterial and insecticidal activities. The volatile constituents are naturally occurring multicomponent combinations of isoprenoids with distinctive odors that are produced by aromatic plants as secondary metabolites. For several cellular processes, including cell division, photosynthesis, growth regulation, and mitochondrial respiration, isoprenoids are physiologically necessary. They are produced from mevalonic acid (MVA), which is the primary source of the active isoprene unit isopentenyl diphosphate, which is a unit of isoprene. The variety of volatile components in this structure has drawn researchers and experts to investigate their significance in relation to industrial and biological activities [6,7]. Throughout history and in all cultures, aromatic herbal plants have been extensively medicinally used to prevent, treat, and control a wide range of ailments [5,8,9,10]. 

Like other natural products that possess wide varieties of biological activities—for example, pomegranate possesses promising antioxidant, antifungal, and hypoglycemic activities [11]—essential oils are known to have a wide range of biological effects, including antiseptic, antifungal, anti-inflammatory, testicle-protective, antibacterial, antioxidant, analgesic, sedative, and anticancer properties [12,13,14,15]. 

EOs are also used to preserve food; eugenol, pinene, limonene, thymol, linalool, farnesyl acetone, and carvone have antimicrobial impacts [16]. Other phenolic elements, such as thymol, have antioxidant properties, and cedrelanol and aromadendrene have an anti-inflammatory effect [16]. Some components of essential oils, such as farnesyl acetone, farnesol, hexadecane, caryophyllene, eicosane, *β*-elemene, farnesyl acetate, *β*-elemene, and *α*-humulene, have been described as cytotoxic substances and have shown promising chemotherapeutic efficacy [17]. Farnesyl acetone has been shown in certain studies to exert cytotoxic and antiproliferative effects on cells, as well as other biological effects, such as insecticidal, sedative, spasmolytic, and antibacterial effects [18,19]. 

Protein kinases (PKs) are a class of enzymes that phosphorylate other proteins involved in signal transmission during cell division [20]. Diseases such as cancer and autoimmune, diabetic, and cardiovascular conditions are caused by mutations and dysregulation of PKs during cell division [21]. Most of the PK inhibitors that have been given approval can interact with the ATP-binding site kinases’ hinge regions. Over the last 40 years, enormous resources have been used by both commercial and academic institutions to assess and define the pathological and physiological roles of PKs in transduction pathways [20]. 

A minor genus in the Asteraceae (Compositae) family, Launaea, has roughly fifty-four species and is found all over the world, but it is most prevalent in Africa, Asia, and the Mediterranean region. The majority of these species are used as insect repellents, lactagogues, hypoglycemics, anti-inflammatory treatments, stomachic treatments, and soporifics in traditional medicines across the globe [22,23,24]. Some Launaea species have also been reported to treat a variety of conditions, including diarrhea, gastric disorders, infected wounds, fever, and hepatic pains. The existence of different metabolites, such as sesquiterpene lactones, flavonoids, triterpenoid saponin, steroids, and coumarins, in addition to essential oils, was discovered through phytochemical analysis of these plants [14,23,25]. 

The phytochemical makeup of EOs from several Launaea species has been the subject of numerous investigations, but the EO from *L. mucronata* has received little attention. The content and cytotoxic potential of the EO produced by *L. mucronata,* which grows in the northern KSA, have also not been investigated. The goal of this investigation was to examine the chemical makeup of the EO produced by *L. mucronata* in relation to the northern KSA region, as well as its bioactivity; this was supported by an in silico analysis of the main elements.

## 2. Results

### 2.1. Essential Oil Compositions

The hydro-distilled volatile components of *L. mucronata* flowers produced a 0.27% weight-per-weight pale yellow fragrant product in the current investigation. The volatile content of a total of 87 individuals (97.98%) was described. The highest percentages of ingredients were 77 light oxygenated compounds (83.83%), 2 monoterpenes (0.04%), and 8 sesquiterpenes (14.81%). First, the majority of the components were determined in the light oxygenated compounds (LOCs), such as 2Z,6E-farnesyl acetone (46.35%), farnesol (10.74%), α-cadinol (2.52%), 5E,9Z-farnesyl acetone (2.22%), 2Z,6Z-farnesal (1.47%), and cedr-8(15)-en-9-ol (1.44%). The next main classes included n-eicosane (10.92%) and cedrane (1.05%), which are sesquiterpenes. Dihydro citronellol acetate (2.35%), which is a light oxygenated compound (LOC), made up the final majority of the compounds. Additionally, included in Table 1 [4,26,27,28,29] is tetrahydro-lavandulol acetate (1.61%).

### 2.2. Antioxidant Assay

DPPH, ABTS, and *β*-carotene assays were used to evaluate the *L. mucronata* EO’s antioxidant properties. Since this EO exhibited significant antioxidant activity, it may be used as an antioxidant agent in medicine (Table 2). According to the DPPH, ABTS, and *β*-carotene assays, respectively, the scavenging power of the *L. mucronata* EO ranged from 30.13 to 65.34, 31.75 to 64.78, and 30.78 to 63.71%. According to the *β*-carotene, ABTS, and DPPH assays, respectively, the values of the standard antioxidant medication t-butyl hydroquinone (TBHQ) varied from 43.35 to 79.11, 42.12 to 78.41, and 42.85 to 78.79% at the same sample concentrations.

### 2.3. Antiproliferative Activity

The cytotoxic potential of the *L. mucronata* EO was evaluated using an MTT assay in triplicate. The assays were conducted on three cell lines (MCF-7, HepG2, and HCT-116). The results are expressed in μg/mL, and strong cytotoxic effects were shown against all the tested cell lines (6.78, 8.45, and 10.24 μg/mL for HepG2, HCT-116, and MCF-7, respectively), in Table 3. In particular, the strong cytotoxic effect against HepG2 may be attributed to the high percentages of farnesol (10.74%), bisabolol (0.84%), calamenene (0.85%), eicosane (10.92%), and farnesyl acetone (46.35%), which have been reported to have cytotoxic effects [30,31,32]. 

### 2.4. Molecular Docking Study

**Figure 1:**
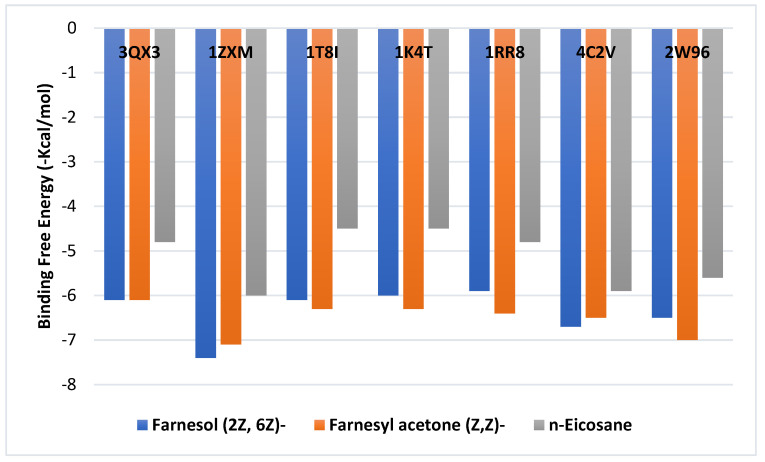
Binding free energy values were calculated using the molecular docking of ligands (farnesol, farnesyl acetone, and eicosane) and receptors (3QX3, 1ZXM, 1T8I, 1K4T, 1RR8, 4C2V, and 2W96).

Figure 1 displays the ability of the ligands to bind to various topoisomerases: topoisomerase I (PDB IDs: 1T8I, 1K4T, and 1RR8), topoisomerase II (PDB IDs: 3QX3 and 1ZXM), aurora B kinases (PDB ID: 4C2V), and CDK4 kinase (PDB ID: 2W96). The binding energies for the ligands on topoisomerase I enzymes were very similar, ranging from −4.5 to −6.3 kcal/mol. The greatest scores for receptors in this range were between −6.3 and −6.4 kcal/mol for farnesyl acetone. An identical pattern was seen when farnesyl acetone docked with CDK4 kinases, recording −7 kcal/mol. Farnesol, on the other hand, displayed a greater affinity for topoisomerase II enzymes (−6.1 and −7.4 kcal/mol) and aurora B kinases (−6.7 kcal/mol). 

**Figure 2:**
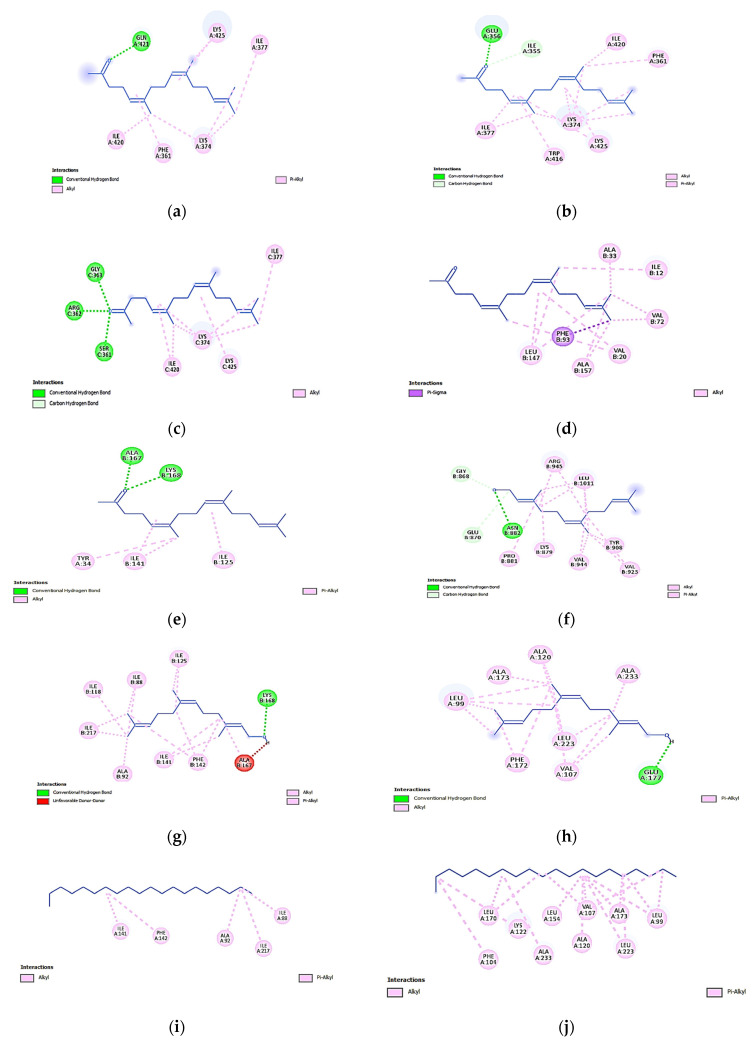
Interactions of farnesyl acetone with topoisomerase I enzymes ((**a**) 1T8I, (**b**) 1K4T, and (**c**) 1RR8), CDK4 kinase ((**d**) 2W96), and topoisomerase II enzymes ((**e**) 1ZXM). Interactions of farnesol with topoisomerase II enzymes ((**f**) 3QX3 and (**g**) 1ZXM) and aurora B kinase ((**h**) 4C2V). Interactions of eicosane with topoisomerase II enzymes ((**i**) 1ZXM) and aurora B ((**j**) 4C2V).

Farnesyl acetone had a higher affinity for attaching to 1RR8 due to the critical conventional hydrogen bonds generated with GLY C:363, ARG C:362, and SER C:361, whereas 1T8I had a higher affinity due to its similarly special bond with GLN A:421 (Figure 2a–c). All the ligands and topoisomerase I enzymes shared alkyl interactions and pi-alkyl bonds; however, they were less effective in terms of binding energy than traditional H-bonds or C-H bonds. In the same scenario, the increased affinity of farnesyl acetone for CDK4 kinases (2W96) (Figure 2d) and topoisomerase II enzymes (1ZXM) (Figure 2e) was due to the strong pi-sigma bond with PHE B:93. Otherwise, farnesol exhibited higher binding energy compared with that of the other ligands when docking with topoisomerase II enzymes (3QX3 and 1ZXM) due to the conventional H-bond and C-H bonds with the ASN B:882, GLY B:368, and GLU B:870 residues for the former enzyme and the conventional H-bond and the unique donor–donor bond with LYS B:168 and ALA B:167 for the latter enzyme (Figure 2f,g). Farnesol and aurora B kinases only displayed one conventional H-bond (4C2V), whereas all the other ligand-to-previous-enzyme connections were created through alkyl and pi-alkyl interactions (Figure 2h). Because of its simple structure and absence of aromaticity, hydroxyl groups, and other functional groups, n-eicosane had a lower binding energy than the other molecules. This may also explain why it had a lower binding affinity. As a result, eicosane had a limited ability to bind to the target enzymes and form a complex (Figure 2i,j).

### 2.5. In Silico ADME Profile

The drug-likeness of farnesol and farnesyl acetone is shown in Table 4 in comparison with that of eicosane. Eicosane had lower lipophilicity, solubility, and TPSA parameters, in addition to a low TPSA value. Farnesol and farnesyl acetone, however, showed better ADME profiles with higher TPSA values (Table 4). The results showed that the investigated compounds were moderately metabolized in the liver, as they only inhibited specific CYP isoforms, as shown in Table 4. Additionally, all the compounds tested were identified as non-Pgp substrates.

## 3. Discussion

### 3.1. Essential Oil Compositions

The yield of *L. mucronata* EOs under investigation was 0.27% *w*/*w*, while that of the Egyptian version of the plant was 0.019 *w*/*w* [4]. The analysis of the Saudi EO resulted in the characterization of 87 components (Table 1), whereas only 50 components were identified in the Egyptian *L. mucronata* EOs [4]. The total content of oxygenated compounds identified in the tested oil sample was 83.5%, while that in the Egyptian plant was 75%.

The main identified components were 2*E*,6*Z*-farnesol, α-cadinol, dihydro citronellol acetate, 5*E*,9*Z*-farnesyl acetone, *n*-hexadecane, tetrahydro lavandulol acetate, 2*Z*,6*Z*-farnesal, *n*-tridecanol, *n*-hexadecanol, and cedrane. 

It was found that the concentrations of *Z*,*Z*-farnesyl acetone, *n*-eicosane, and Cedr-8(15)-en-9-α-ol were significantly different (46.53, 10.92, and 1.44%, respectively) from those in the Egyptian plant (3.95, 1.38, and 2.23%, respectively). The significant variation between the Saudi and Egyptian plants regarding their yield and chemical composition might be attributable to the different localities and environmental conditions. 

### 3.2. Antioxidant Assay

The ability of a medication or phytochemical to shield from or even stop the oxidation of the human body by dangerous radicals is known as antioxidant activity [33,34]. When the EO of *L. mucronata* was compared with that of TBHQ as a reference, the EO showed significant antioxidant activity in all three assays. According to the DPPH, ABTS, and *β*-carotene assays, the evaluated EO sample’s scavenging power ranged from 30.13 to 65.34, 31.75 to 64.78, and 30.78 to 63.71%, respectively. According to the *β*-carotene, ABTS, and DPPH assays, respectively, the results for TBHQ ranged from 43.35 to 79.11, 42.12 to 78.41, and 42.85 to 78.79% (Table 2). The results of all the assays were consistent with each other. The complex blend of the *L. mucronata* EO’s contents, which were mostly light oxygenated components (farnesol, farnesyl acetone, 1-terpineol, and ocimenone), may be responsible for its potent antioxidant activity [35]. Our results were found to be in good agreement with those of Thejanuo et al. [36]. Additionally, the antioxidant activity of the investigated sample was greater than that reported in various Launaea species growing in Egypt [4]. Moreover, the inhibition percentage of the EO’s scavenging activity (64–79) at a concentration of 80 mg/mL was greater than that obtained from a methanolic extract of the same plant collected from ArAr, Saudi Arabia (50%) [37]. Therefore, rather than using its extract, *L. mucronata* EO might be introduced as a food preservative. 

### 3.3. Antiproliferative Activity

An MTT assay was used to assess the cytotoxic capabilities of the *L. mucronata* EO against several cell lines (MCF-7, HepG2, and HCT-116). For HepG2, HCT-116, and MCF-7, respectively, the results showed substantial cytotoxic potential (6.78, 8.45, and 10.24 g/mL) (Table 3), which might be attributable to the presence of high concentrations of farnesyl acetone (46.35%), eicosane (10.92%), farnesol (10.74%), calamenene (0.85%), and bisabolol (0.84%), which have been reported to have cytotoxic effects [30,31,32]. Our results were found to be in good agreement with those of Palanisamy et al. [38,39].

### 3.4. Molecular Docking

The primary bioactive components of the EO (farnesol, farnesyl acetone, and *n*-eicosane) were subjected to docking studies for topoisomerase I, topoisomerase II, aurora B kinases, and CDK4 kinase. Certain compounds had a greater affinity than others for binding to the residues of the target enzymes due to the crucial conventional bonds that formed with the target residues (Figure 1 and Figure 2). Farnesyl acetone had the highest binding energies with topoisomerase I enzyme receptors (−6.3 to −6.4 kcal/mol) and CDK4 kinases (−7 kcal/mol). Farnesol was shown to have the highest affinity (−6.1 to −7.4 kcal/mol) for topoisomerase II enzymes and aurora B kinases. On the other hand, when farnesol docked with the topoisomerase II enzymes 3QX3 and 1ZXM, it showed higher binding energy than that of the other ligands. Due to its straightforward structure and lack of functional groups, which may favor its interactions with target enzymes, n-eicosane displayed the lowest binding energy. As a result, eicosane had a limited ability to bind to the target enzymes (Figure 1 and Figure 2). The molecular docking analysis validated the *L. mucronata* EO’s promising antiproliferative action, which may be explained by the compound’s farnesyl acetone and farnesol content, as these demonstrated increased binding affinity for the target enzymes [40,41,42].

### 3.5. In Silico ADME

SwissADME was used to evaluate the drug-likeness of the compounds based on guidelines considering various descriptors, such as molecular weight, lipophilicity, solubility, flexibility, and topological surface area. Lipophilicity and solubility are essential parameters for drug absorption, while the number of rotatable bonds should be less than 10 for drug-likeness, according to Veber’s rule [40,41,42]. Unsaturation in compounds can improve receptor–ligand complementarity. The results of the comparison of the drug-likeness of farnesol and farnesyl acetone with that of eicosane agreed with previous findings (Table 4). Eicosane had lower binding energies than those of farnesol and farnesyl acetone, which was in agreement with the violations of the lipophilicity, solubility, and TPSA parameters, as shown in Table 4. This was due to its nonpolar structure, which increased its hydrophobicity in relation to that of the other compounds studied. In addition, its low TPSA value indicated its low polarity, leading to poor oral absorption and membrane permeation. Farnesol and farnesyl acetone, on the other hand, showed better ADME profiles by incorporating polar moieties, such as hydroxyl and carbonyl groups, which increased their TPSA values (Table 4). Therefore, adding polar functional groups is crucial for increasing bioavailability [40,41]. 

It was necessary to perform further analyses to predict the metabolic rates of the compounds under investigation—specifically, as cytochrome P450 (CYP) enzyme inhibitors and as Pgp substrates. The cytochrome P450 (CYP) enzyme superfamily plays a crucial role in hepatic drug metabolism, while the Pgp substrate is a type of drug efflux transporter that helps limit cellular uptake and increase the elimination of drugs through excretion organs. 

## 4. Materials and Methods

### 4.1. Plant Material

*Launaea mucronata* (Forssk.) Muschl. flowers were collected in April 2019 on the campus of Jouf University in Al-Jouf, Saudi Arabia. Mr. Hamidan Hasan, M.Sc. (Camel and Range Research Center, Al-Jouf, KSA) successfully verified the authenticity of the plant sample. The College of Pharmacy at Jouf University in Saudi Arabia has a voucher sample (71-CPJU) that is archived and kept there.

### 4.2. Extraction of the Volatile Constituents

In April 2019, 350 g of fresh flowers were obtained from *L. mucronata* and cleaned with ordinary water; then, the volatile oil was hydro-distilled with a Clevenger apparatus using the conventional extraction method. The distillate was separated from the aqueous phase using a separating funnel with a volume of 500 mL. By adding NaCl (salting out), the complete amount of oil was exhausted. Dichloromethane was then used to extract the remaining soluble ingredients from the aqueous phase. After being dehydrated with anhydrous Na_2_SO_4_, the mixed extracts were filtered with Whatman filter paper (WHA1001025, Zhejiang, China). The hydro-distilled extract that was produced (0.27% *w*/*w*) was a yellowish oily liquid with a pleasant smell. Until further investigation, it was kept in airtight, dry, sealed vials at a temperature of 2–4 °C [43,44].

### 4.3. Gas Chromatography and Gas Chromatography–Mass Spectrometry (GC-FID and GC-MS)

For the measurement of the volatile components, an Agilent gas chromatograph apparatus (model 6890, Folsom, CA, USA) equipped with an FID (flame ionization detector) at 70 eV and an HP-5ms capillary column (120 m × 0.25 mm) was used. The feed ratio for the carrier gas (He) was set to 20 cm/sec, the injector and detector temperatures were set to 250 °C, and the oven temperature was varied from 60 to 240 °C and maintained for 10 min. 

Agilent Technologies’ model 7890B GC (Folsom, CA, USA) in conjunction with a 7000D GC/TQ, GC/MS, and 7693A autosampler was utilized to analyze the volatiles and identify them. Ionization occurred at 70 eV with a 120 m × 0.25 mm i.d. HP-5ms column. The injector and detector were kept at a constant temperature of 250 °C during the entire experiment, which was run at a constant velocity of 30 cm/s for the carrier gas (He). The run continued for 50 min at the preset temperature (60–240 °C) with a 3 °C/min temperature increase that was maintained until the end of the run. By injecting the sample with a solution series of homologous n-hydrocarbons (C8–C26) under identical pre-existing conditions, values of Kovat’s index were determined. By comparing the fragmentation patterns with those from NIST data, published data, and Kovat’s retention indices of authentic components, the hydro-distilled constituents were determined. This study reports the composition of volatile components as a comparative percentage of the entire peak region. 

### 4.4. Antioxidant Activity

#### 4.4.1. DPPH Radical Scavenging Assay

DPPH, a stable radical that has a dark purple color and can absorb UV radiation of up to 518 nm in wavelength, was used. When antioxidants are present, DPPH takes an electron, stabilizing the radical. After that, the solution is decolored, and the absorbance at the maximum wavelength of 518 nm decreases [37]. By measuring the decrease in absorbance and comparing the IC_50_ with that of recognized antioxidants, such as tert-butylhydroquinone (TBHQ), the antioxidant capabilities can be determined. The absorbance was measured using a UV spectrophotometer (HP 8452, UV-VIS, Bothell, WA, USA) [35]. The following calculation was used to calculate the antioxidants’ ability to scavenge the radical as a percentage of inhibition:

% inhibition = [(A _control_ − A _sample or standard_)]/(A _control_) × 100

#### 4.4.2. *β*-Carotene/Linoleic acid Bleaching Assay

A previously discussed linoleic acid/*β*-carotene scheme was used to determine the antioxidant capacity of the hydro-distilled extract of *L. mucronata* flowers. Utilizing TBHQ as a positive control, the absorbance was measured at a maximum wavelength of 471 nm. The following equation was used to compute the inhibition percentage of bleaching (I bleaching percentage) [45]:

[Absorbance (after 2 h of experiment)/Initial absorbance] × 100 = I bleaching%

#### 4.4.3. ABTS Free Radical Assay

Following the Witayapan method with a few minor modifications, a stock solution of ABTS was diluted in methanol, and a substance called 2,2’-azino-bis [3-ethylbenzothiazoline-6-sulfonic acid] was used to determine the scavenging capabilities of the hydro-distilled components of *L. mucronata* flowers. Then, 2.45 mM K_2_S_2_O_8_ was used to oxidize the solution, resulting in the formation of pre-formed ABTS radical monocation. The mixture was held at 25 °C for 12 h in a dark environment, and the absorbance was measured at a maximum of 733 nm. The equation specified in the description of the DPPH assay was used to determine the antioxidant inhibition percentage in the ABTS assay [35].

### 4.5. Antiproliferative Activity

The MCF-7 (Michigan Cancer Foundation-7), HepG2 (human hepatocellular carcinoma), and HCT-116 (human colorectal carcinoma) cell lines were used in this investigation. Fetal bovine serum (10%) (FBS-Gibco, Sigma Aldrich, St. Louis, MI, USA) was added to the cell lines, which were purchased from the Regional Center for Mycology and Biotechnology at Al-Azhar University in Cairo, Egypt [46,47]. Humidified CO_2_ (5%) was used to maintain the cells at 36 °C. Using the MTT assay in accordance with the Lang and Denizot method, the in vitro antiproliferative capabilities of the volatile contents of *L. mucronata* flowers were assessed. A maximum of 598 nm was used to measure the decrease in the level of MTT with respect to formazan blue within the cells.

### 4.6. Docking Study

The Protein Data Bank (PDB) (https://www.rcsb.org/, accessed on 17 December 2021) was used to obtain the crystal structures of topoisomerase I enzymes (PDB IDs: 1T8I, 1K4T, and 1RR8), topoisomerase II enzymes (PDB IDs: 3QX3 and 1ZXM), aurora B kinases (PDB ID: 4C2V), and CDK4 kinases (PDB ID: 2W96) [48]. Avogadro software (Version 1.2.0) was used to optimize the 3D structures of the ligands (farnesol, farnesyl acetone, and eicosane), and these were retrieved from the PubChem database (http://pubchem.ncbi.nlm.nih.gov/, accessed on 17 December 2021) [48]. On 17–18 December 2021, the web-based program CB-DOCK (http://clab.labshare.cn/cb-dock/php/, accessed on 17 December 2021) was used to execute blind docking. The input files were reviewed by CB-Dock after submission, and OpenBabel and MGLTools translated them into pdbqt format. The protein cavities were then predicted using CB-Dock, which also determined the locations and dimensions of the top N (n = 5 by default) cavities. The docking of all the centers, sizes, and pdbqt files was performed using AutoDock Vina. The findings were presented following the computation of N rounds [36]. Using the Discovery Studio program (Version 21.1.0.20298), interaction and visualization profiles were created for the best-docked complexes [36].

### 4.7. In Silico ADME Properties

The SwissADME server (http://www.swissadme.ch/) (accessed on 16 September 2023) from the Swiss Institute of Bioinformatics was used to evaluate all the ligands’ in silico ADME profiles. The SMILES notations were generated and submitted to the server during ligand preparation for the ADME evaluation [35].

### 4.8. Statistical Analysis

The data (means ± SD) were analyzed using one-way ANOVA followed by the Tukey–Kramer test. The level of significance was set at a probability of less than 0.05. GraphPad Prism 8 software was used for statistical analysis.

## 5. Conclusions

An analysis of the EO from *Launaea mucronata* resulted in the characterization of 87 components, the majority of which were represented by 2*Z*,6*E*-farnesyl acetone (46.35%), 2*E*,6*Z*-farnesol (10.74%), and *n*-eicosane (10.92%). The EO showed promising antioxidant and antiproliferative activities in comparison with the standards used. The docking study supported the oil’s antiproliferative effect. The overall conclusion is in support of the safe application of the EO from *L. mucronata* at low concentrations in aromatherapy as a strong antioxidant agent for food preservation.

## Figures and Tables

**Table 1:** Chemical composition of *L. mucronata* EO according to GC-MS analysis.

Peak No.	Rel. Comp. %	Calculated * KI	KI Data	Compound Name	Compounds’ Class	Identification Methods
1	0.02	1024	1023–1027	Trimethyl benzene (1,2,4)	M	MS&KI
2	0.02	1029	1028–1034	*β*-Phellandrene	M	MS&KI
3	0.02	1035	1033–1038	2-acetyl-5-methyl-furan	LOC	MS&KI
4	0.06	1038	1035–1041	5-methyl-hexanoic acid	LOC	MS&KI
5	0.15	1048	1045–1048	*γ*-hexalactone	LOC	MS&KI
6	0.02	1068	1065–1069	2-methyl-benzaldehyde	LOC	MS&KI
7	0.04	1086	1085–1089	3-methyl-1,2-cyclohexanedione	LOC	MS&KI
8	0.02	1099	1095–1099	2-nonanol	LOC	MS&KI
9	0.06	1107	1104–1109	2,6-dimethyl phenol	LOC	MS&KI
10	0.05	1109	1108–1112	*cis*-rose oxide	LOC	MS&KI
11	0.02	1115	1114–1118	Endo-fenchol	LOC	MS&KI
12	0.1	1121	1119–1123	exo-fenchol	LOC	MS&KI
13	0.02	1124	1122–1125	Myrcenol	LOC	MS&KI
14	0.51	1130	1127–1131	Octyl formate	LOC	MS&KI
15	0.34	1132	1132–1136	1-terpineol	LOC	MS&KI
16	0.14	1139	1138–1140	*trans*-pinocarveol	LOC	MS&KI
17	0.05	1144	1142–1145	*cis*-Pinene hydrate	LOC	MS&KI
18	0.05	1147	1144–1147	Camphor	LOC	MS&KI
19	0.02	1150	1146–1151	Camphene hydrate	LOC	MS&KI
20	0.07	1155	1153–1156	Isobutyl hexanoate	LOC	MS&KI
21	0.05	1156	1155–1159	Nerol oxide	LOC	MS&KI
22	0.02	1160	1160–1163	*cis*-dihydro-*β*-terpineol	LOC	MS&KI
23	0.15	1165	1164–1168	2*E*-nonenol	LOC	MS&KI
24	0.02	1172	1170–1174	Octanoic acid	LOC	MS&KI
25	0.07	1176	1172–1177	*cis*-pyranoid linalool oxide	LOC	MS&KI
26	0.1	1184	1181–1184	Thuj-3-en-10-al	LOC	MS&KI
27	0.05	1187	1185–1188	neoiso-menthol	LOC	MS&KI
28	0.04	1194	1193–1197	*cis*-piperitol	LOC	MS&KI
29	0.02	1200	1199–1201	*cis*-4-caranone	LOC	MS&KI
31	0.05	1212	1211–1215	Iso-dihydro carveol	LOC	MS&KI
32	0.05	1223	1221–1225	Methyl-2*E*-nonenoate	LOC	MS&KI
33	0.71	1238	1237–1239	*E*-ocimenone	LOC	MS&KI
34	0.68	1247	1246–1251	Ethyl-oct-2*E*-enoate	LOC	MS&KI
35	0.05	1264	1263–1266	*cis*-chrysanthenyl acetate	LOC	MS&KI
36	1.61	1271	1268–1272	tetrahydro-lavandulol acetate	LOC	MS&KI
37	0.15	1276	1274–1277	dihydro-linalool acetate	LOC	MS&KI
38	0.28	1281	1279–1282	3*Z*-hexenyl valerate	LOC	MS&KI
39	0.02	1283	1280–1284	*cis*-verbenyl acetate	LOC	MS&KI
40	0.15	1288	1286–1289	2-ethyl-endo-fenchol	LOC	MS&KI
41	0.07	1301	1298–1302	*trans*-dihydro-*α*-terpinyl acetate	LOC	MS&KI
42	0.33	1304	1303–1307	Undecanal	LOC	MS&KI
43	0.25	1313	1312–1317	Citronellic acid	LOC	MS&KI
44	2.35	1321	1319–1322	Dihydro citronellol acetate	LOC	MS&KI
45	0.02	1354	1351–1355	Thymol acetate	LOC	MS&KI
46	0.1	1360	1360–1363	2*E*-Undecenal	LOC	MS&KI
47	0.15	1389	1388–1391	2-dodecanone-methyl decyl ketone	LOC	MS&KI
48	0.12	1391	1390–1392	3-Dodecanone	LOC	MS&KI
49	0.02	1394	1391–1394	*β*-elemene	S	MS&KI
50	0.05	1401	1398–1402	*β*-longipinene	S	MS&KI
51	0.35	1408	1406–1409	Dodecanal	S	MS&KI
52	1.05	1442	1440–1444	Cedrane	S	MS&KI
53	0.31	1446	1446–1450	Bakerol	LOC	MS&KI
54	0.25	1455	1453–1456	Geranyl acetone	LOC	MS&KI
55	0.85	1491	1490–1494	10,11-epoxy-calamenene	LOC	MS&KI
56	0.45	1506	1203–1507	*E*,*E-α*-Farnesene	S	MS&KI
57	0.05	1554	1553–1557	Thymohydro quinone	LOC	MS&KI
58	0.22	1568	1567–1569	2*E*-Tridecen-1-al	LOC	MS&KI
59	1.15	1573	1570–1573	*n*-Tridecanol	LOC	MS&KI
60	1.85	1600	1558–1601	*n*-Hexadecane	S	MS&KI
61	0.05	1604	1602–1606	Ledol	LOC	MS&KI
62	0.12	1606	1605–1609	Geranyl isovalerate	LOC	MS&KI
63	0.05	1611	1608–1611	Dodecyl acetate	LOC	MS&KI
64	0.17	1615	1613–1616	*cis*-isolongifolanone	LOC	MS&KI
65	0.02	1617	1615–1619	Davanol D1	LOC	MS&KI
66	0.36	1628	1627–1629	2-(3-oxobutyl)-isomenthone	LOC	MS&KI
67	0.07	1650	1648–1651	*β*-eudesmol	LOC	MS&KI
68	1.44	1653	1650–1653	Cedr-8(15)-en-9-*α*-ol	LOC	MS&KI
69	0.25	1654	1652–1655	*α*-eudesmol	LOC	MS&KI
70	2.52	1655	1654–1657	*α*-cadinol	LOC	MS&KI
71	0.02	1656	1655–1659	Geranyl valerate	LOC	MS&KI
72	0.22	1667	1666–1669	14-hydroxy-(*Z*)-caryophyllene	LOC	MS&KI
73	0.41	1675	1674–1678	*Z*-nerolidyl acetate	LOC	MS&KI
74	1.47	1683	1682–1686	2*Z*,6*Z*-farnesal	LOC	MS&KI
75	0.84	1686	1685–1689	*α*-bisabolol	LOC	MS&KI
76	10.74	1713	1709–1713	2*E*,6*Z*-farnesol	LOC	MS&KI
77	0.75	1715	1712–1716	14-hydroxy-*α*-Humulene	LOC	MS&KI
78	46.35	1860	1857–1882	*Z*,*Z*-farnesyl acetone	LOC	MS&KI
79	0.12	1855	1853–1858	Cyclopentadecanolide	S	MS&KI
80	0.02	1862	1859–1863	Eudesm-7(11)-en-4-ol, acetate	LOC	MS&KI
81	0.8	1865	1864–1867	Homoisobaeckeol	LOC	MS&KI
82	0.5	1870	1869–1873	2,7(14),10-bisabolatrien-1-ol-4-one	LOC	MS&KI
83	0.02	1880	1878–1882	*α*-chenopodiol	LOC	MS&KI
84	1.12	1885	1883–1887	*n*-hexadecanol	LOC	MS&KI
85	2.22	1891	1891–1896	5*E*,9*Z*-farnesyl acetone	LOC	MS&KI
86	0.37	1900	1898–1902	Dihydro-columellarin	LOC	MS&KI
87	10.92	2001	2000–2004	*n*-eicosane	S	MS&KI

* KI: Kovat’s index; the calculated KI data were compared with those obtained from the literature, as well as documented websites dealing with the KI ranges of volatiles. Rel. comp. (relative composition) %: comparative percentage of the entire peak region. MS: tentative characterization through comparison with NIST mass spectra library data. M: monoterpenes. S: sesquiterpenes. LOC: light oxygenated compound.

**Table 2:** Antioxidant potential of the volatile oil of *L. mucronata*.

Sample Concentration (µg/mL)	% Inhibition by DPPH		% Inhibition by ABTS		% Inhibition by *β*-Carotene/Linoleic Acid	
	EO	TBHQ	EO	TBHQ	EO	TBHQ
20	30.13 ± 1.8	42.85 ± 1.9	31.75 ± 1.8	42.12 ± 1.8	30.78 ± 1.9	43.35 ± 1.8
40	42.65 ± 1.9	65.02 ± 1.8	41.26 ± 1.9	64.72 ± 1.9	43.32 ± 1.9	66.01 ± 2.0
60	53.71 ± 1.9	72.15 ± 2.1	51.25 ± 1.9	71.84 ± 2.0	52.16 ± 2.1	71.65 ± 2.1
80	65.34 ± 2.1	78.79 ± 2.0	64.78 ± 2.2	78.41 ± 2.2	63.91 ± 2.1	79.11 ± 2.1

Values are presented as the average of experiments performed in triplicate ± the standard deviation (*p* < 0.005).

**Table 3:** Antiproliferative activity of the *L. mucronata* essential oil.

Compounds	IC_50_ ± SD (μg/mL) ^a^		
	MCF-7	HepG2	HCT-116
EO	10.24 ± 2.14	6.78 ± 1.82	8.45 ± 1.64
Doxorubicin	0.81 ± 0.83	0.85 ± 0.48	0.78 ± 0.63

^a^ Cell proliferation was determined using an MTT assay. IC_50_ ± SD or IC_50_ values in µg/mL after 48 h of incubation. The values are the average of three independent experiments. SD: standard deviation. MTT: 3-(4,5-dimethylthiazol-2-yl)-2,5-diphenyltetrazolium bromide. IC_50_: concentration that achieved 50% inhibition of the proliferation response exhibited in the specified cell lines without treatment.

**Table 4:** In silico ADME properties of farnesol, farnesyl acetone, and eicosane according to SwissADME.

ADME Properties	Identifier	Farnesol	Farnesyl Acetone	Eicosane
Physicochemical Properties	Molecular weight	222.37	262.43	282.55
	No. rotatable bonds	7	9	17
	TPSA	20.23Å	17.07 Å	0.00 Å
Lipophilicity	iLOGP	3.71	3.67	5.64
	XLOGP3	5.42	5.56	10.45
	WLOGP	4.40	5.77	8.05
	MLOGP	3.86	4.50	7.38
	SILICOS-IT	4.21	5.69	7.98
Water Solubility	ESOL	−4.17	−4.38	−7.05
	Log S	−5.60	−5.68	−10.40
	SILICOS-IT	−3.15	−4.47	−7.94
Pharmacokinetics	GI absorption	High	High	Low
	BBB permeant	Yes	No	No
	P-gp substrate	No	No	No
	CYP1A2 inhibitor	Yes	Yes	Yes
	CYP2C19 inhibitor	No	No	No
	CYP2C9 inhibitor	Yes	Yes	No
	CYP2D6 inhibitor	No	No	No
	CYP3A4 inhibitor	No	No	No
	Log K_p_	−3.81 cm/s	−3.95 cm/s	−0.6 cm/s
Drug-likeness	Lipinski	Yes	Yes	Yes
	Ghose	Yes	No	No
	Veber	Yes	Yes	No
	Egan	Yes	Yes	No
	Muegge	No	No	No
	Bioavailability score	0.55	0.55	0.55
Medicinal Chemistry	PAINS	0 alert	0 alert	0 alert
	Brenk	1 alert	1 alert	0 alert

## Data Availability

Not applicable.

## Sample Availability

Samples are available from the authors.
